# Cortical vestibular-visual interactions and cross-modal plasticity–adaptive neural processes for stable perception

**DOI:** 10.3389/fncir.2026.1876597

**Published:** 2026-07-03

**Authors:** Huizhe Sun, Ziyi Tan, Fu Zeng, Aihua Chen

**Affiliations:** Key Laboratory of Brain Functional Genomics (Ministry of Education), East China Normal University, Shanghai, China

**Keywords:** congruent and opposite neurons, cross-modal recalibration, multisensory integration, sensory conflict, vestibular cortex, visual motion

## Abstract

The integration of vestibular and visual signals is essential for spatial orientation, self-motion perception, and postural stability. However, sensory conflicts and environmental changes demand adaptive neural mechanisms to maintain perceptual reliability. Cross-modal plasticity provides a key mechanism for this adaptation by dynamically recalibrating sensory representations in response to discordant or altered inputs. In this review, we summarize the cortical pathways underlying vestibular and visual processing, discuss how multisensory integration and segregation are implemented through distinct neuronal populations and computational principles. We further emphasize recalibration as a core adaptive mechanism that operates across multiple timescales-from gradual adjustments to persistent cue discrepancies to rapid updates shaped by recent sensory history and prior decisions. By synthesizing behavioral and neural evidence, we reveal differential adaptive responses across multisensory cortical areas and propose visual-vestibular recalibration as a fundamental principle of multisensory plasticity, with broad implications for understanding brain function and developing translation approaches for sensory and balance disorders.

## 1 Introduction

Accurate perception of self-motion is essential for navigation, spatial orientation, and behavioral interaction with the environment. To achieve this, the brain integrates information from multiple sensory modalities, among which visual and vestibular signals are particularly critical. Visual cues, particularly optic flow, provide rich information about heading direction, movement speed and spatial structure of the environment (Gibson, 1950; Warren and Hannon, 1988). However, visual motion signals are inherently ambiguous, because similar retinal motion patterns can arise from self-motion, object motion, or eye movements (Warren and Hannon, 1990). Consequently, visual signals alone are insufficient for reliable self-motion estimation and must be interpreted in conjunction with non-visual information, most importantly vestibular signals (Telford et al., 1995; Angelaki and Cullen, 2008).

Vestibular signals originate from the semicircular canals and otoliths, which encode head rotation and linear acceleration with high temporal precision. However, vestibular responses are primarily driven by changes in motion and provide limited information during sustained constant-velocity movement (Merfeld et al., 1999; Angelaki, 2004). In contrast, visual motion signals can continuously represent self-motion through optic flow and provide stable spatial references to the external environment. Thus, visual and vestibular systems contribute complementary aspects of self-motion processing: vision where we are relative to the environment, while the vestibular system tells us how our head is moving from moment to moment (Lappe et al., 1999; Angelaki and Cullen, 2008; DeAngelis and Angelaki, 2012; Angelaki, 2014). The integration of these signals allows the brain to construct coherent and stable representations of movement through space.

Importantly, visual-vestibular interaction involves more than simple fusion. Because signals from different modalities may originate from either a common source or independent causes, the brain faces a fundamental computational challenge: determining when cues should be integrated and when they should remain segregated. Under congruent conditions, combining visual and vestibular signals according to their reliability improves perceptual precision and reduces uncertainty (Gu et al., 2008; Fetsch et al., 2009, 2011; Chen et al., 2013). In contrast, when discrepancies arise - such as during object motion, sensory perturbations, or artificial cue conflicts – mandatory fusion would produce inaccurate percepts. In these situations, the nervous system must preserve cue separation to infer distinct causal sources (de Winkel et al., 2017; Acerbi et al., 2018; Dokka et al., 2019). Therefore, adaptive self-motion perception depends on a dynamic balance between multisensory integration and separation.

Moreover, optimal integration and separation are not static processes. In daily life, the relationship between visual and vestibular signals changes with development, aging, injury, and altered environments such as virtual reality or corrective devices (Aller and Noppeney, 2019; Jones et al., 2019; Rohlf et al., 2020). To maintain perceptual stability and behavioral accuracy, the brain must continuously adjust sensory weighting and internal mappings over time (Fetsch et al., 2009; Rohlf et al., 2020). Cross-modal recalibration is one form of this adjustment (Zaidel et al., 2011, 2013). It operates across multiple timescales, from rapid changes driven by recent sensory history to slower changes produced by persistent discrepancies (Zaidel et al., 2011, 2013; Shalom-Sperber et al., 2022). Through recalibration, the nervous system maintains flexible yet stable multisensory representations in changing environments.

In recent years, converging evidence from psychophysics, neurophysiology, neuroimaging, and computational modeling has significantly advanced our understanding of visual-vestibular processing (Hou and Gu, 2020; Noel and Angelaki, 2022; Zeng Z. et al., 2023). These studies reveal that multisensory cortical areas contain heterogeneous neuronal populations that support distinct computational roles, including cue integration, causal inference, and adaptive recalibration. At the same time, computational frameworks such as Bayesian inference and reliability-weighted coding have provided normative accounts of how the brain flexibly combines and updates sensory information.

In this review, we first summarize the cortical pathways for vestibular and visual processing, then examine how integration and separation are supported by distinct neuronal populations and computational principles. Finally, we focus on recalibration as an adaptive process that links sensory conflict to longer-term changes in multisensory processing. Rather than introducing a new formal computation model, we present the Integration-Separation-Recalibration (ISR) framework as an organizing synthesis of existing behavioral, neural, and computational findings. A central idea of this synthesis is that integration and segregation determine how sensory information is interpreted on a given trial, whereas recalibration updates the priors, mappings, and reliabilities that shape future interpretations. Thus, perception and learning form a continuous closed loop, allowing the brain to maintain stable yet flexible visual–vestibular representations despite changing sensory environments.

## 2 Cortical vestibular and visual processing

Understanding visual-vestibular interactions in self-motion perception first requires mapping the cortical pathways that carry these signals and identifying where they converge. Vestibular and visual motion information originate from different sensors and are processed through partially distinct cortical streams (Angelaki, 2014; Hou and Gu, 2020). These circuits networks provide the anatomical substrate not only for multisensory integration, but also for experience-dependent plasticity that stabilizes perception over time.

### 2.1 Vestibular pathways

Vestibular signals originate in the semicircular canals and otolith organs, which encode angular and linear acceleration, respectively (Fernandez and Goldberg, 1971, 1976a,b,c; Goldberg and Fernandez, 1971). These signals are transmitted via the vestibular nerve to the vestibular nuclei (VN) in the brainstem (Highstein and Holstein, 2006). From the VN, vestibular information ascends through vestibulo-thalamic pathways and reaches multiple cortical targets, forming a distributed vestibular cortical network (Lopez and Blanke, 2011). Subcortical structures also contribute to vestibular processing. The superior colliculus receives vestibular inputs and integrates them with visual and auditory signals to generate rapid orienting behaviors, such as turning the head and eyes toward a perceived motion or sound sources (Maeda et al., 1979; Meredith and Stein, 1986; Wallace et al., 1996). The thalamus, in turn, acts as a relay station, transmitting vestibular signals from the brainstem to multiple cortical areas (Meng et al., 2007; Meng and Angelaki, 2010), including parieto-insular vestibular cortex (Lopez and Blanke, 2011).

**FIGURE 1:**
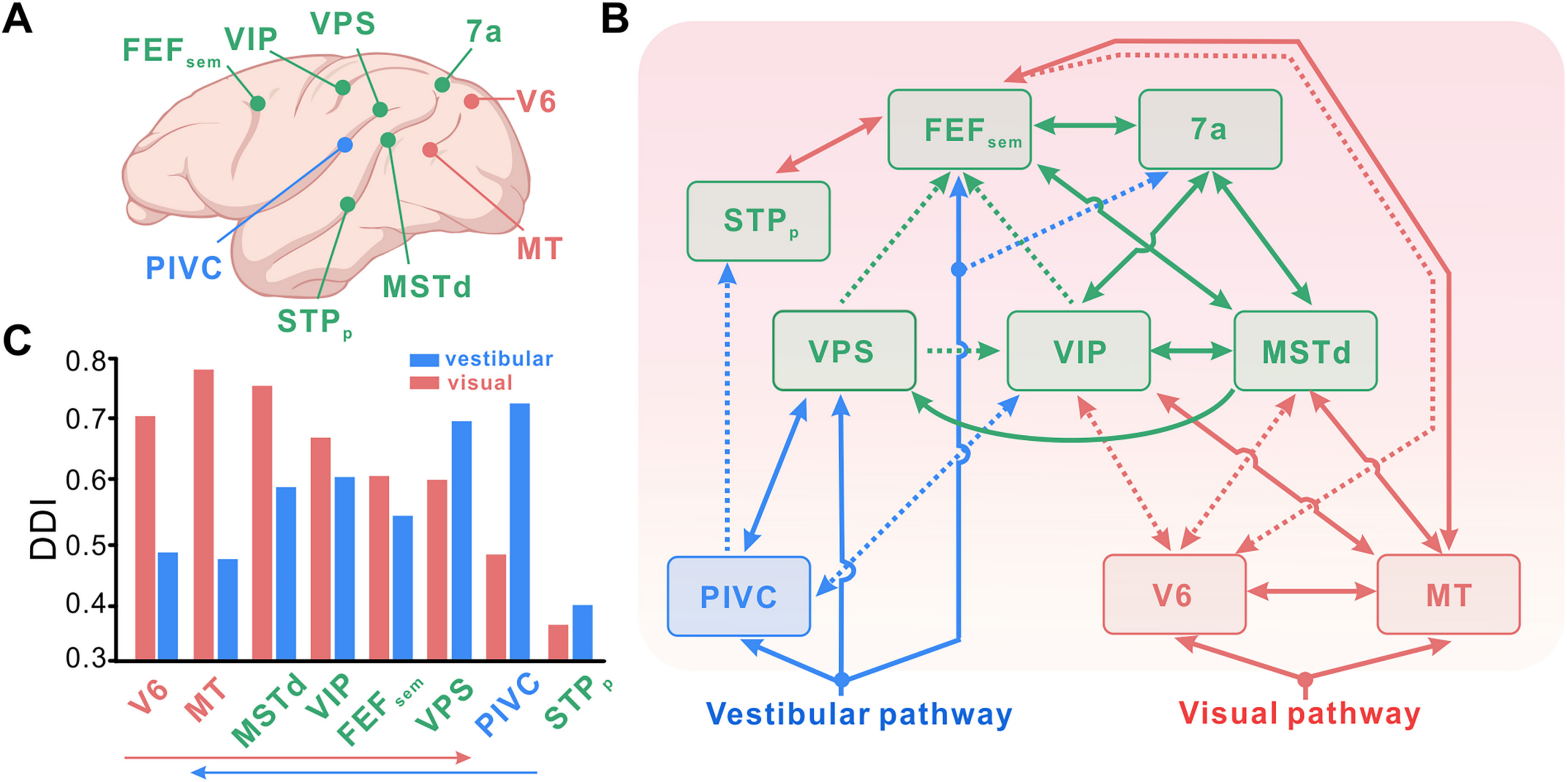
Vestibular and visual pathways in self-motion perception. **(A)** Schematic lateral view of the macaque cortex showing major cortical regions involved in visual-vestibular processing during self-motion. **(B)** Proposed cortical network for heading perception during self-motion. Based on neuronal recordings from macaques undergoing whole-body translation, this schematic summarizes possible information flow among cortical regions involved in visual–vestibular integration. Arrows indicate the likely direction of signal transmission derived from prior electrophysiological evidence. Vestibular-dominant areas and pathways are shown in blue, visual-dominant in red, and multisensory integration hubs in green. MSTd, dorsal portion of medial superior temporal area; PIVC, parieto-insular vestibular cortex; VPS, visual posterior sylvian area; VIP, ventral intraparietal area; FEFsem, smooth eye movement region of the frontal eye field; V6, area V6; MT, middle temporal area; STPp, posterior superior temporal polysensory area. Anatomical connections and proposed information flow are adapted from Felleman and Van Essen (1991) and Lopez and Blanke (2011). **(C)** Spatial tuning strength quantified by a direction discrimination index (DDI). DDI value ranges from 0 to 1, with 0 indicating no selectivity and 1 indicating high selectivity. Red: DDI values measured under the visual conditions. Blue: DDI values measured under the vestibular conditions. Source values for the DDI comparison are summarized in Table 1.

A key cortical hub within this network is the parieto-insular vestibular cortex (PIVC), which is widely regarded as a core vestibular region (Grusser et al., 1990). Most PIVC neurons show limited or no responses to visual motion, suggesting that this area provides a predominantly vestibular signal for downstream multisensory regions (Chen et al., 2010). As shown in Figures 1A,B, PIVC is connected with several cortical areas involved in self-motion processing (Felleman and Van Essen, 1991; Lopez and Blanke, 2011), including the dorsal medial superior temporal area (MSTd; Duffy, 1998; Page and Duffy, 2003; Gu et al., 2006, 2007), the ventral intraparietal area (VIP; Bremmer et al., 2002a,b; Schlack et al., 2002; Zhang and Britten, 2010; Chen et al., 2011c), the visual posterior sylvian area (VPS; Chen et al., 2011b), the smooth eye movement area of the frontal eye field (FEFsem; Gu et al., 2016), the posterior superior temporal polysensory area (STPp; Zhao et al., 2021), and area 7a (Avila et al., 2019).

**TABLE 1:** Direction discrimination index values and source references.

Brain region	DDI		N	Reference
	Visual	Vestibular		
V6	0.72 ± 0.010	0.49 ± 0.007	106	Fan et al., 2015
MT	0.78 ± 0.013	0.47 ± 0.0009	47	Chowdhury et al., 2009
MSTd	0.76 ± 0.01	0.59 ± 0.01	340	Gu et al., 2006; Takahashi et al., 2007
VIP	0.66 ± 0.01	0.61 ± 0.01	452	Chen et al., 2011c
FEF_*sem*_	0.62 ± 0.0086	0.55 ± 0.0075	229	Gu et al., 2016
VPS	0.60 ± 0.01	0.69 ± 0.01	166	Chen et al., 2011b
PIVC	0.49 ± 0.009	0.74 ± 0.02	288	Chen et al., 2010
STP_*p*_	0.40 ± 0.12	0.35 ± 0.11	191	Zhao et al., 2021

DDI values are reported as mean ± SEM for visual and vestibular conditions in each cortical area. N indicates the number of recorded neurons included in the corresponding source study.

In humans, converging evidence from functional neuroimaging, lesion studies, and electrical stimulation supports the existence of a distributed vestibular cortical network spanning posterior insular, parietal opercular, retroinsular, and temporo-parietal regions, rather than a single primary vestibular cortex (Lopez et al., 2012; Klingner et al., 2013; Kirsch et al., 2016; Raiser et al., 2020). Within this network, area OP2 in the posterior parietal operculum has emerged as a candidate hub for vestibular processing and is often considered a possible functional homolog macaque PIVC (zu Eulenburg et al., 2012; Ibitoye et al., 2023), although this correspondence remains debated. Importantly, human studies indicate extensive interactions between vestibular and visual-motion processing networks, supporting the integration of optic-flow and vestibular signals during self-motion perception as well as the resolution of sensory conflicts when these cues are inconsistent (Brandt et al., 2002; Lopez and Blanke, 2011). These findings suggest that visual-vestibular processing in humans, as in non-human primates, relies on distributed multisensory cortical circuits rather than a single convergence site.

Beyond self-motion perception, vestibular signals also contribute to predictive processing that separates self-generated from externally generated motion (Cullen, 2019) and interact with hippocampal and parahippocampal circuits involved in navigation and memory (Hitier et al., 2014). Overall, the vestibular pathway provides rapid body-centered motion signals to multiple cortical targets.

### 2.2 Visual motion pathways

Visual motion information is transmitted from the retina through the lateral geniculate nucleus (LGN) to primary visual cortex (V1), and then along the dorsal stream to motion-sensitive regions such as the middle temporal area (MT) and MSTd (Maunsell and Van Essen, 1983). Neurons in V1 and MT typically have relatively small receptive fields and encode local motion components (Hubel and Wiesel, 1974, 1977; Albright, 1984; Albright and Desimone, 1987). In contrast, MSTd neurons integrate motion signals over much larger portions of the visual field (Duffy and Wurtz, 1991; Duffy, 1998). This large-scale integration supports the extraction of global optic flow patterns - such as radial expansion, contraction, and rotation - which are critical for heading perception during self-motion (Britten and van Wezel, 1998; Duffy, 1998).

Psychophysical studies have shown that optic flow based self-motion perception depends not only on retinal motion, but also on extraretinal signals related to eye movements (Warren and Hannon, 1988; Royden et al., 1992). Consistent with this, MSTd appears to lie at a stage where local motion signals are transformed into more global representations of self-motion. As shown in Figures 1A,B, visual motion signals from MT and V6 can reach MSTd and other multisensory areas, while MSTd further projects to multisensory regions such as VIP (Chen et al., 2011c) and VPS (Chen et al., 2011b), providing a direct route by which optic-flow information can converge with vestibular signals (Boussaoud et al., 1990). In this way, visual motion signals can be processed at successive stages before being combined with vestibular information during heading perception.

### 2.3 Multisensory convergence

Visual and vestibular signals converge most prominently in MSTd, VIP, and the PIVC/VPS complex (Duffy, 1998; Bremmer et al., 2002b; Chen et al., 2010, 2011b; 2016). However, these regions differ in the relative strength of visual and vestibular input. MSTd is often described as more visually driven (Gu et al., 2006, 2008), PIVC as more vestibular-dominant (Chen et al., 2010, 2016), and VIP as a site of strong, balanced multisensory convergence (Chen et al., 2011a,c, 2013). This pattern suggests that the cortical self-motion network is heterogeneous rather than uniform.

As illustrated in Figure 1, MSTd occupies a key intermediate stage for visual and vestibular convergence. Many MSTd neurons respond to both optic flow and inertial motion (Duffy, 1998; Gu et al., 2006). Some show similar preferred directions across the two modalities (congruent neurons), whereas others show mismatched tuning (opposite neurons). Correlations between MSTd activity and discrimination performance also support a role in heading decisions, particularly through congruent neuronal populations (Gu et al., 2008). Causal studies further suggest that MSTd contributes more strongly to visual heading judgments than to vestibular heading judgments: microstimulation and reversible inactivation strongly bias or impair visual heading perception, but have weaker effects on vestibular judgments (Britten and van Wezel, 1998; Gu et al., 2012). In humans, vestibular influences have also been reported in MT+ (hMT/hMST), where galvanic vestibular stimulation (GVS) evokes vestibular responsiveness, especially in anterior hMST (hMSTa), paralleling vestibular responses observed in macaque MSTd (Smith et al., 2012).

Visual posterior sylvian area represents a distinct convergence site with strong sensitivity to both modalities, but with a notable prevalence of opposite tuning (Chen et al., 2011b). This property has been linked to encoding cross-modal mismatch and may support conflict-dependent computations rather than direct cue fusion. In humans, the posterior insular cortex (PIC) has been proposed as a possible counterpart of macaque VPS (Sunaert et al., 1999), although this correspondence remains tentative and is based mainly on functional similarities. Neuroimaging and electrophysiological studies suggest that PIC responds to both vestibular and visual stimulation, supporting the idea that it is an important locus for visual-vestibular interaction (Cardin and Smith, 2010; Frank et al., 2014; Frank and Greenlee, 2018; Wirth et al., 2018).

Ventral intraparietal area is another major convergence area. It receives both visual and vestibular inputs and has been linked to multisensory heading discrimination (Zhang and Britten, 2010; Chen et al., 2011c,^2013^). Analysis of the direction preferences of VIP neurons revealed both congruent and opposite neurons, similar to what has been described in MSTd (Schlack et al., 2002; Chen et al., 2013). Compared with MSTd, VIP activity is more strongly influenced by decision-related factors (Zaidel et al., 2017; Chen et al., 2021). However, causal inactivation studies suggest that VIP is not strictly required for basic heading discrimination under standard conditions (Chen et al., 2016). Taken together, these findings indicate that while VIP is not a mandatory hub for simple perceptual decisions, it may play a distinct role in more complex or flexible aspects of behavior – such as linking multisensory representations to action selection, adjusting cue integration based on context demands, or supporting adaptive changes when sensory relationships are altered (Zaidel et al., 2021; Zeng F. et al., 2023).

Additional regions contribute to this distributed network. FEFsem neurons show selectivity to both visual and vestibular inputs, their established role in smooth eye movement suggests that their multisensory signals may subserve oculomotor coordination during self-motion, rather than perceptual readout *per se* (Gu et al., 2016). Area 7a is vestibular-dominant and primarily encoded the magnitude, rather than the direction, of self-motion speed (Avila et al., 2019). STPp is comparatively visual-dominant and appears to contribute less to heading perception than MSTd, VIP, or VPS (Zhao et al., 2021). The overall anatomical connectivity and information flow across these cortical nodes are summarized in Figure 1, which highlights how vestibular-dominant, visual-dominant, and multisensory areas form an interconnected hierarchy supporting self-motion perception.

The functional differences among these areas are further reflected in the direction discrimination index (DDI) shown in Figure 1C. The DDI comparison reveals a clear functional gradient across the network: visual-dominant regions such as MT and V6 show stronger visual than vestibular tuning, PIVC shows stronger vestibular than visual tuning, and multisensory regions such as MSTd, VIP, FEFsem, and VPS exhibit substantial tuning under both conditions.

Recent rodent studies further expand our understanding of visual-vestibular convergence. Vestibular stimulation in rodents evokes widespread activity across thalamic, limbic, and cortical regions, indicating that vestibular signals influence a broad forebrain network rather than a limited set of classical vestibular areas (Rancz et al., 2015). Consistent with this view, mouse primary visual cortex receives real-time head-motion information, including direction, velocity, and acceleration signals, through thalamocortical and corticocortical pathways (Bouvier et al., 2025). These findings suggest that vestibular-related self-motion signals can influence cortical processing at much earlier stages than previously appreciated, extending beyond higher-order multisensory areas.

Together, these findings suggest that the cortical self-motion network is not a uniform integration system, but a functionally heterogeneous hierarchy. Visual-dominant areas (e.g., MT and V6) primarily encode optic-flow information, whereas vestibular-dominant regions (e.g., PIVC) provide robust inertial-motion signals. Between these extremes lie multisensory convergence areas with distinct functional biases: MSTd is relatively visual-dominant, VPS is relatively vestibular-dominant, and VIP exhibits more balanced visual and vestibular representations. Recent rodent studies further broaden this framework by showing that vestibular-related signals may also influence early stages of visual cortical processing. This distributed organization provides a substrate not only for multisensory integration, but also for cue separation, sensory conflict detection and adaptive recalibration, which are discussed in later sections.

### 2. 4 Computational implications

The vestibular and visual systems differ substantially in their coding properties, implying that their combination requires flexible computational strategies. Otolith-related pathways encode gravito-inertial and linear acceleration, whereas semicircular-canal-related pathways convey information about angular head motion. Importantly, these signals undergo substantial transformations within downstream vestibular pathways. In the vestibular nuclei (VN), many neurons exhibit frequency-dependent response dynamics, such that their activity may reflect mixtures of acceleration-, velocity-, and position-related signals depending on motion conditions (Bush et al., 1993; Angelaki et al., 2000). In contrast, visual motion pathways extract self-motion information from optic flow distributed across the visual field, providing rich spatial and environmental context but requiring additional processing to distinguish self-motion from object motion or eye movements. These complementary properties motivate adaptive multisensory computations: the brain integrates visual and vestibular cues when they are reliable and consistent, yet maintains cue separation when sensory signals conflict.

A central idea from earlier work is that cue combination depends on relative reliability (van Beers et al., 1996; Ernst and Banks, 2002; Gu et al., 2008; Morgan et al., 2008; Fetsch et al., 2009; Ohshiro et al., 2011, 2017; Prsa et al., 2012). When visual and vestibular signals are aligned, weighting each cue according to its reliability can improve the precision of heading estimates (Gu et al., 2008; Fetsch et al., 2009). However, self-motion perception also requires mechanisms for source separation and conflict resolution. When the two signals diverge, the system may need to reduce the influence of one cue, preserve separate estimates, or otherwise limit fusion in order to maintain perceptual stability (Acerbi et al., 2018; Dokka et al., 2019).

This balance between integration and separation also sets the stage for recalibration. Persistent mismatches can drive experience-dependent changes in sensory weights or internal mappings, allowing later perception to remain stable under altered conditions (Zaidel et al., 2011, 2013; Tong et al., 2018; Aller and Noppeney, 2019). In this way, the cortical pathways described above provide not only the anatomical routes for immediate multisensory processing, but also the network substrate on which adaptive plasticity can reshape how self-motion cues are combined across different contexts.

## 3 Dynamic integration and segregation of visual–vestibular signals

During self-motion perception, the brain must determine when combining cues improves estimation and when keeping them separate is more appropriate. This section reviews behavioral evidence for cue integration and segregation, then summarizes neural signatures in key cortical areas, and finally outlines computational models that link behavior to neural mechanisms.

### 3.1 Behavioral evidence

**FIGURE 2:**
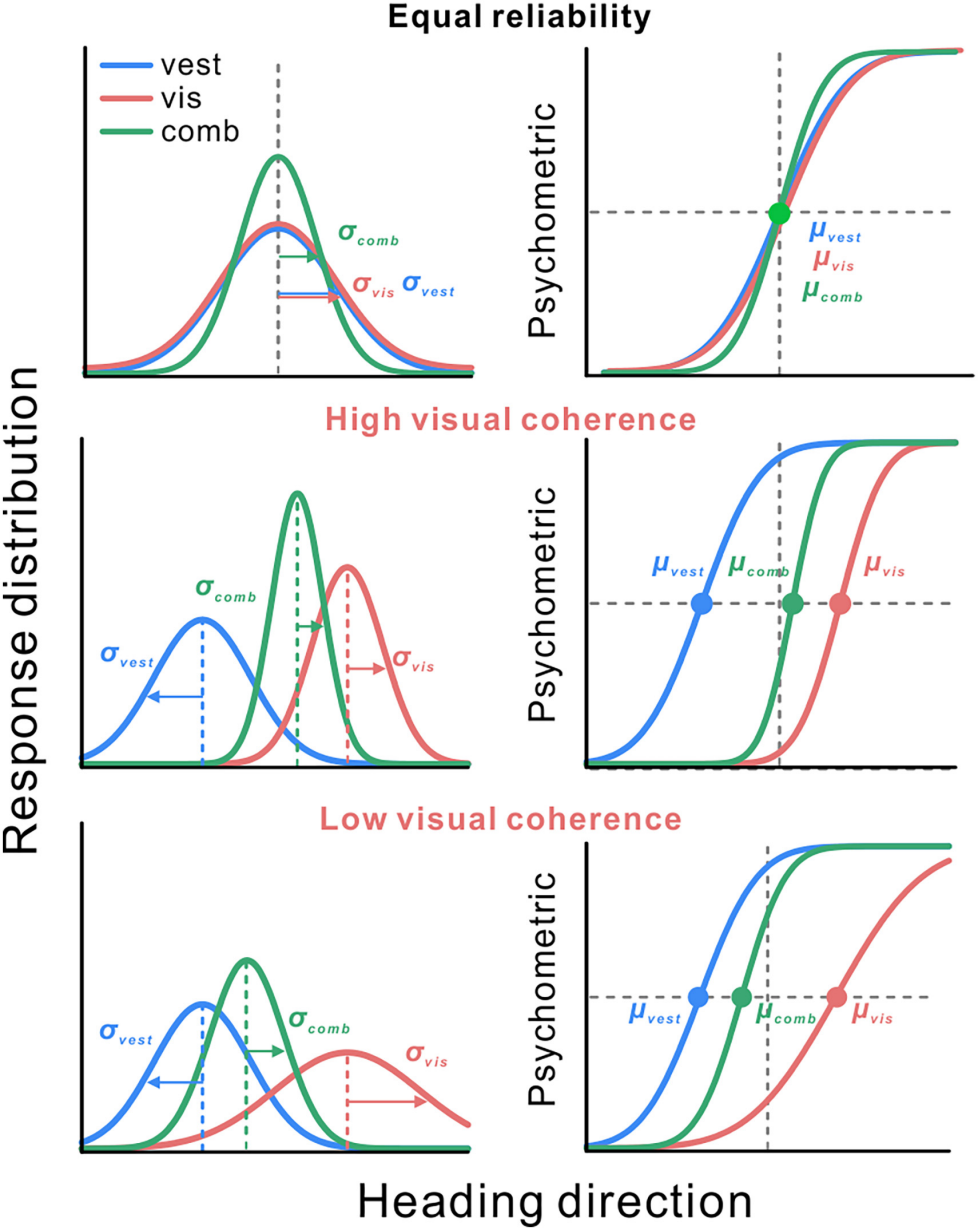
Behavioral signatures of reliability-weighted cue combination in visual–vestibular heading perception. Left panels show schematic probability distributions of vestibular, visual, and combined heading estimates. Right panels show schematic psychometric functions from a heading discrimination task. **(Top)** When visual and vestibular cues are consistent and equally reliable, their combination reduces uncertainty and improves perceptual precision. **(Middle)** Under cue conflict with high visual reliability (high coherence), the combined estimate is biased toward the visual cue. **(Bottom)** Under cue conflict with low visual reliability (low coherence), the combined estimate shifts toward the vestibular cue.

When visual and vestibular cues are consistent, behavior typically reflects reliability-weighted integration. In heading discrimination tasks, combined visual and vestibular stimulation leads to lower perceptual thresholds than either cue alone, consistent with the idea that pooling independent noisy estimates improves precision (Gu et al., 2008; Fetsch et al., 2009; Butler et al., 2010). The contribution of each cue depends on its reliability: when visual motion becomes less reliable, observers place greater weight on vestibular signals, whereas more reliable optic flow leads to stronger visual weighting (Fetsch et al., 2009; Butler et al., 2010; Figure 2).

However, heading perception in natural settings frequently involves cue discrepancies. Under conditions of visual and vestibular conflict, behavior reveals two interacting phenomena: (i) systematic biases in perceived heading and (ii) context-dependent cue weighting. For small cue disparities, observers often show partial fusion, producing intermediate percepts biased toward the other modality (Kording et al., 2007; de Winkel et al., 2017; Acerbi et al., 2018; Dokka et al., 2019; Figure 2). As disparity increases, integration weakens: perceptual biases become non-linear, cue weights shift asymmetrically, and behavior may resemble the maintenance of separate estimates rather than a single fused percept (Acerbi et al., 2018).

These patterns indicate that cue combination is governed not only by reliability, but also by an implicit estimate of whether the signals share a common origin. This is captured by causal-inference frameworks, in which the brain computes the probability that visual and vestibular cues arise from a common cause (Kording et al., 2007; Shams and Beierholm, 2010). High inferred common-cause probability promotes integration, whereas low probability favors segregation. This framework accounts for the continuous transition between fusion and separation observed in behavior, without requiring discrete switching rules.

Importantly, integration behavior is further modulated by context. Task demands, feedback, and prior experience can all bias how cues are combined. For example, tasks emphasizing perceptual reporting versus conflict detection can lead to different integration strategies (Zaidel et al., 2013; van Atteveldt et al., 2014; Acerbi et al., 2018). Trial history and priors also influence cue weighting even when sensory reliability is unchanged (Fetsch et al., 2011; Rohlf et al., 2020), indicating that the brain maintains dynamic beliefs about both cue reliability and causal structure. Together, these findings show that multisensory behavior reflects flexible, inference-driven processing, rather than fixed integration rules.

### 3.2 Neural substrates of multisensory integration and conflict

Neurophysiological studies in macaques have identified key cortical substrates underlying this flexibility, particularly in areas such as MSTd and VIP. Neurons in these regions often respond to both visual and vestibular stimuli, but exhibit distinct patterns of cross-modal tuning that reveal how integration and segregation are implemented at the population level (Gu et al., 2008; Fetsch et al., 2011). A central organizational principle is the coexistence of two functional neuronal populations: congruent neurons, whose preferred headings are aligned across visual and vestibular modalities; Opposite neurons, whose preferred directions differ substantially (Gu et al., 2008).

**FIGURE 3:**
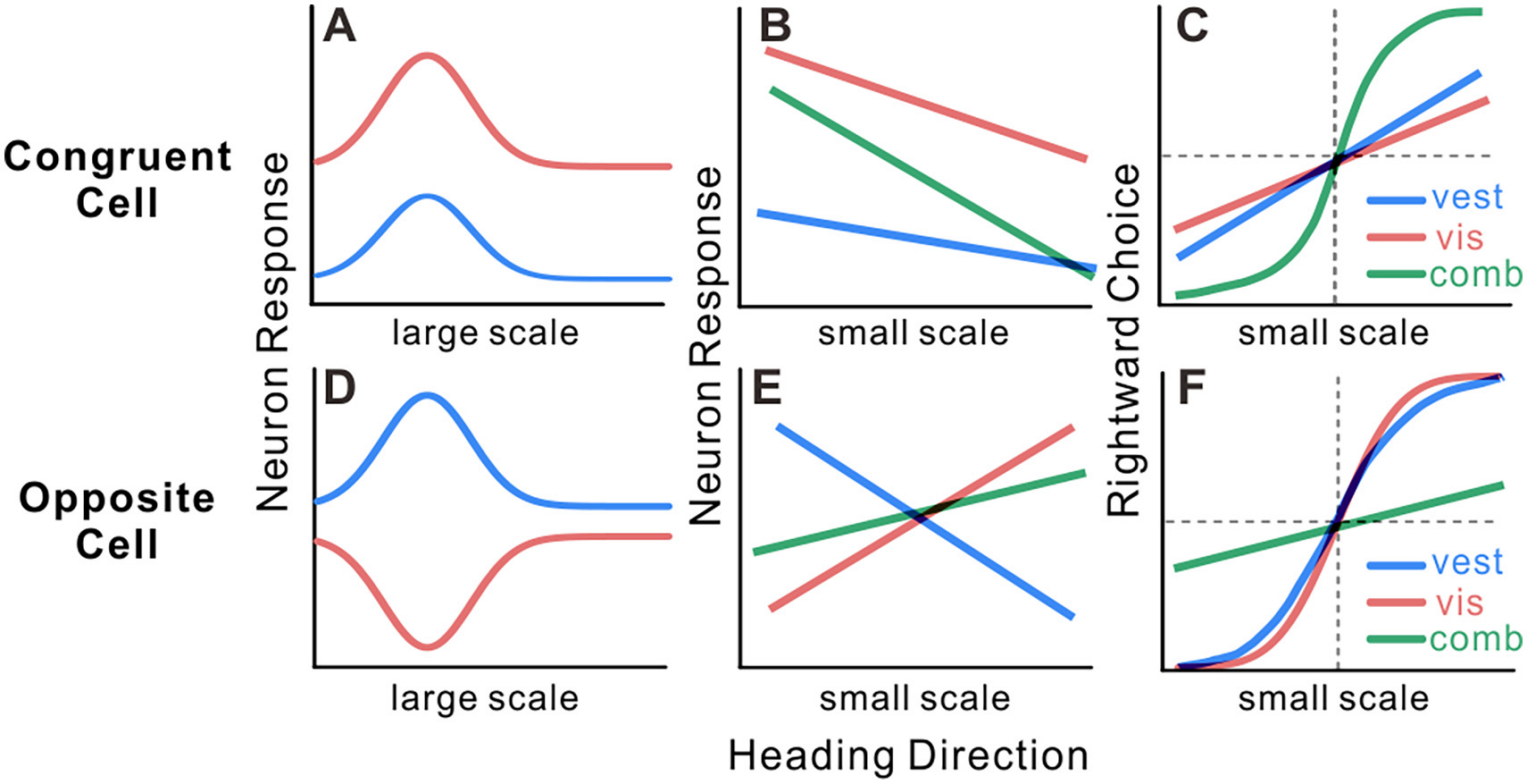
Neural response patterns of congruent and opposite cells during visual–vestibular heading tasks and their relationship to perceptual readouts. **(A–C)** Example congruent cell: tuning curves for vestibular (blue) and visual (red) cues alone **(A)**, as well as combined stimulation **(**green, **B)**, showing enhanced responses when cues are aligned. Corresponding psychometric functions **(C)** reveal improved behavioral performance under combined stimulation. **(D–F)** Example opposite cell: vestibular (blue) and visual (red) tuning exhibit opposing preferences **(D)**, leading to reduced or non-additive responses under combined stimulation **(**green, **E)** and weakened cue integration in the corresponding psychometric curve **(F)**.

These populations differ systematically in their responses to multisensory stimulation. Congruent neurons often show enhanced sensitivity under combined conditions, with neuronal thresholds lower than single-cue conditions, closely paralleling behavioral improvements predicted by optimal cue integration (Gu et al., 2008; Fetsch et al., 2011; Figures 3A–C). Similar patterns have been reported in area VIP, where congruent neuronal activity also followed predictions of optimal multisensory integration models (Chen et al., 2013). By contrast, opposite neurons often show reduced or non-additive responses under cue combination, together with weaker directional sensitivity (Gu et al., 2008; Chen et al., 2013; Figures 3D–F). Rather than contributing directly to cue fusion, these neurons are thought to encode cross-modal mismatch or conflict, providing signals that may support segregation or influence causal inference.

Neural responses are also dynamically modulated by cue reliability. Manipulations of visual coherence systematically alter the relative influence of visual and vestibular inputs on neuronal activity, mirroring behavioral reweighting (Fetsch et al., 2011). Across broader stimulus conditions, multisensory responses in MSTd are often well described by weighted linear combinations of unimodal responses, with weights typically below one, indicating subadditive integration (Morgan et al., 2008; Zhao et al., 2023).

Together, these findings suggest that multisensory processing is not implemented by a single canonical computation. Instead, it emerges from the interaction of distinct neuronal subpopulations with complementary roles: congruent neurons support cue fusion, whereas opposite neurons encode structured mismatch signals that can guide conflict-dependent processing and inference about causal structure.

### 3.3 Computational principles: from cue combination to causal inference

**FIGURE 4:**
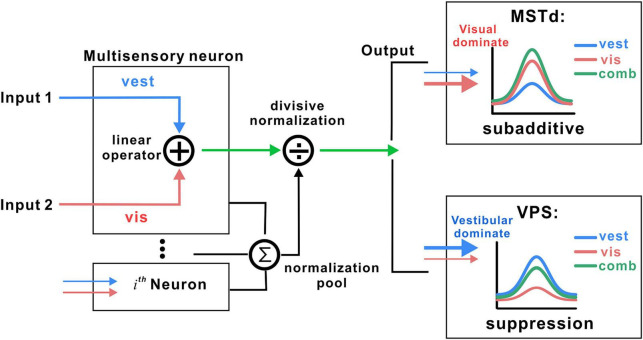
Computational mechanisms of vestibular and visual signal integration across MSTd and VPS. In the MSTd, multisensory neurons linearly integrate vestibular (blue) and visual (red) inputs, with visual signals typically dominating. The integrated signals are subsequently subject to divisive normalization via a normalization pool, resulting in a subadditive combined response (green), where the response to multisensory inputs is weaker than the sum of unimodal responses. In the VPS, vestibular inputs are dominant, and divisive normalization causes the suppression of the combined response relative to the vestibular response alone.

Computational models provide a principled bridge between behavioral cue-combination rules and the neural operations that could implement them (Beck et al., 2008; Ohshiro et al., 2011, 2017). At the neural level, divisive normalization offers a canonical computation for multisensory integration (Ohshiro et al., 2011, 2017). In this framework, neuronal responses are scaled by the overall activity of a population, naturally producing subadditive responses when multiple inputs are combined. Such normalization mechanisms can account for both reliability-dependent weighting and the compressive nature of multisensory responses observed in areas such as MSTd and VPS (Morgan et al., 2008; Zhao et al., 2023; Figure 4).

At the algorithmic level, reliability-weighted integration models describe how cues can be combined into a single estimate with weights proportional to cue reliability when they are assumed to share a common source (Ernst and Banks, 2002; Butler et al., 2010). However, these models are limited in that they do not account for behavior under substantial cue conflict. Bayesian causal inference models extend this framework by incorporating a judgment about whether cues share a common cause (Kording et al., 2007; Shams and Beierholm, 2010; Rohe and Noppeney, 2015b). This model predicted a continuum of behaviors, from near-optimal fusion at small disparities, to partial fusion at intermediate disparities, to segregation-like behavior when disparities are large, depending on both cue reliability and disparity.

At the neural level, causal inference is increasing viewed as a graded and hierarchical computation rather than a categorical switch between integration and segregation. Neuroimaging studies suggest that segregated sensory representations, fused estimates, and causal-inference signals may emerge at different stages of the cortical processing, with computations evolving dynamically from sensory to association and frontal cortices (Rohe and Noppeney, 2015a; Cao et al., 2019; Rohe et al., 2019);. Within this framework, congruent and opposite neurons provide complementary evidence streams for multisensory inference. Congruent neurons support common-cause interpretations by encoding consistent visual and vestibular heading information, whereas opposite populations encode cue-disparity signals between modalities (Zhang et al., 2019). Such disparity signals may facilitate segregation when visual and vestibular cues are unlikely to arise from a common source, but may also contribute to distinguishing self-motion from independently moving objects or other external motion sources. Thus, opposite neurons should not be viewed solely as segregation signals, but more generally as neural representations of cross-modal mismatch that can support multiple perceptual functions depending on behavioral context.

Recent theoretical developments further emphasize that integration and segregation need not be mutually exclusive outcomes (Rohe and Noppeney, 2015a; Acerbi et al., 2018; Rohe et al., 2019). Continuous-mixture models allow partial integration and segregation to coexist, weighted by the inferred probability of a common cause (Kording et al., 2007). Similarly, probabilistic population coding frameworks propose that neural populations encode probability distributions over sensory variables, uncertainty, and causal structure (Ma et al., 2006; Beck et al., 2008). Recurrent neural network models provide a dynamical implementation of these computations (Cao et al., 2019; Rohe et al., 2019), while predictive-coding and active-inference frameworks offer a broader perspective in which integration, segregation, and recalibration emerge from hierarchical prediction-error minimization (Friston, 2005, 2012; Yamashita et al., 2013).

Recent rodent studies extend these principles beyond classical primate multisensory areas. In the mouse retrosplenial cortex, angular head velocity is represented through vestibular-dependent multisensory signals that are refined by visual input during navigation (Keshavarzi et al., 2022). In mouse primary visual cortex, visual flow is combined with vestibular and locomotor signals, allowing self-generated and externally generated motion to be represented differently (Velez-Fort et al., 2025). These findings suggest that visual-vestibular computations can emerge across multiple levels of the cortical hierarchy.

In summary, visual-vestibular perception relies on a hierarchy of computations that extends from reliability-weighted cue combination to probabilistic causal inference. Together, these mechanisms enable flexible transitions between cue integration and cue segregation according to sensory context. However, they do not by themselves explain how perception remains stable when cue relationships change over longer timescales. Addressing this requires adaptive processes that update sensory mappings and internal beliefs-processes collectively referred to as recalibration, which we discuss in the next section.

## 4 Cross modal recalibration: linking inference and plasticity

While integration and separation operate on rapid, moment-to-moment timescales, the brain must also maintain perceptual stability across longer periods during which the relationship between sensory cues may change. Persistent discrepancies between vestibular and visual cues cannot be resolved solely through trial-by-trial inference. Instead, they drive recalibration – a process that updates internal models, sensory weights, and cross-modal mapping to ensure that future perception remains accurate (Shimojo and Shams, 2001; Lewald, 2002; Wozny and Shams, 2011; Zaidel et al., 2011). Crucially, recalibration is not merely a consequence of sensory conflict, but a computational mechanism that links short-term inference with long-term plasticity. In this sense, it closes the loop between perception and learning, allowing the system to adapt to both transient and sustained changes in the environment.

### 4.1 Behavioral evidence: multi-timescale adaptation

Behavioral studies show that visual-vestibular recalibration operates across multiple timescales and is shaped by both sensory statistics and task structure (Zaidel et al., 2011, 2013; Shalom-Sperber et al., 2022).

**FIGURE 5:**
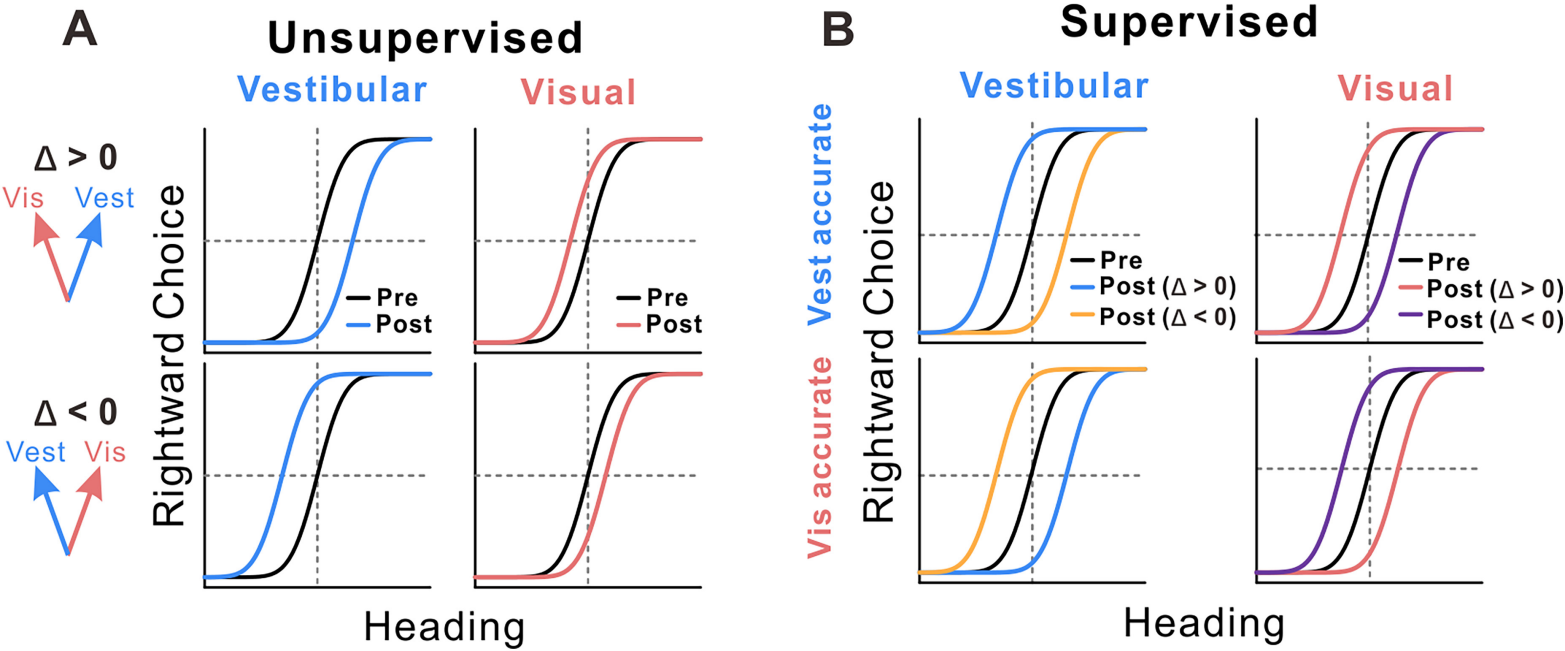
Computational model of recalibration processes in multisensory perception. **(A)** Unsupervised recalibration. The black curve indicates psychometric performance before recalibration. After recalibration, vestibular (blue) and visual (red) heading estimates are shifted. When Δ > 0, vestibular and visual estimates shift rightward and leftward, respectively; when Δ < 0, vestibular and visual estimates shift leftward and rightward, respectively. Psychometric functions exhibit opposite shifts for the two modalities. **(B)** Supervised recalibration. The top and bottom panels correspond to conditions in which the “vestibular-accurate” and “visual-accurate” cues are designated, respectively. Here, “accurate” refers to the consistency between the cue and external feedback. In both conditions, the psychometric curves shift in the same direction, moving toward the modality identified as accurate.

Long-term recalibration emerges when observers are exposed to sustained discrepancies between visual and vestibular cues over many trials. Under such conditions, perceived heading gradually shifts, reflecting updates to internal sensory mappings (Zaidel et al., 2011). In the absence of external feedback, recalibration is unsupervised: both visual and vestibular estimates shift in opposite directions, reducing the discrepancy between them. Notably, vestibular recalibration is often larger in magnitude, suggesting asymmetric plasticity across modalities (Zaidel et al., 2011; Figure 5A). When external feedback is provided, recalibration becomes supervised. In this case, both modalities tend to shift toward the feedback-defined “correct” reference, effectively aligning perception with an external standard rather than internal consistency (Zaidel et al., 2013; Figure 5B). These two modes-unsupervised and supervised-highlight that recalibration can be driven either by internal conflict minimization or by external error correction.

In contrast, short-term recalibration operates over much shorter timescales, often within a few trials. Recent stimulus history bias subsequent perception, even in the absence of persistent discrepancies (Shalom-Sperber et al., 2022). These rapid effects reflect a form of dynamic updating that integrates recent sensory evidence, prior expectations, and possibly recent choices.

Whether these fast and slow effects reflect distinct recalibration mechanisms remains an open question. One possibility is that multiple adaptive processes operate in parallel, with fast mechanisms tracking recent sensory history and slower mechanisms accumulating evidence over longer periods. Alternatively, both effects may arise from a common adaptive updating process, such as a state-space or Kalman-filter-like mechanism, in which the effective learning rate varies according to sensory uncertainty, prediction errors, and the inferred stability of the environment. From this perspective, apparent timescales may reflect different operating regimes of a shared computational process rather than fundamentally separate mechanisms.

Together, these findings suggest that visual-vestibular recalibration is dynamically regulated by recent sensory history, accumulated cross-modal discrepancies, feedback, and environmental statistics. Understanding how these factors interact to maintain perceptual stability requires identifying the neural circuits and computational mechanisms that support adaptive updating, which we discuss in the following sections.

### 4.2 Neural mechanisms: distributed and heterogeneous plasticity

Neural evidence indicates that recalibration is implemented across a distributed network, rather than localized a single area (Zaidel et al., 2021; Zeng F. et al., 2023). Importantly, different regions exhibit distinct recalibration profiles, suggesting functional specialization across the hierarchy.

During unsupervised recalibration, neural changes are heterogeneous across areas. In MSTd, both visual and vestibular tuning curves shift in directions consistent with their respective perceptual recalibrations. In vestibular-dominant regions such as PIVC, vestibular tuning shifts align with behavioral changes, whereas visual effects are less prominent due to weak visual responses. In contrast, VIP exhibits a distinct pattern: both visual and vestibular tuning tend to shift in accordance with vestibular perceptual recalibration, even when visual perception shifts in the opposite direction (Zeng F. et al., 2023). These dissociations suggest that recalibration is not a simple sensory-level adjustment, but reflects area-specific transformations of multisensory representations. Lower-level areas may encode modality-specific recalibration, whereas higher-level regions integrate these changes into a unified perceptual or decision-related framework.

During supervised recalibration, neural signatures differ again. Feedback-dependent shifts are strongly expressed in VIP, where neuronal tuning tracks behavioral recalibration, but are largely absent in MSTd (Zaidel et al., 2021). This pattern suggests that higher-order multisensory areas are more sensitive to task context and external feedback, consistent with their role in linking perception to decision-making and learning. More generally, these findings connect visual-vestibular recalibration to the wider literature on perceptual decision-making. Parietal and prefrontal circuits are known to encode sensory evidence, choices, and task-dependent variables, while perceptual-history studies demonstrate that recent stimuli, choices, and feedback can bias subsequent perception (Gold and Shadlen, 2007; Fischer and Whitney, 2014; Freedman and Assad, 2016). Thus, some rapid recalibration-like effects may partly reflect decision-history signals, whereas slower forms of recalibration may involve more persistent modifications of sensory mappings, cue weights, or internal models.

These cortical effects should be considered within a broader vestibular-cerebellar-cortical network. The vestibulo-cerebellum plays a central role in vestibular processing and adaptive motor learning, with vestibulo-ocular reflex (VOR) adaptation providing a classical example of cerebellum-dependent recalibration (Robinson, 1976; Lisberger et al., 1984; Boyden et al., 2004). In addition, the nodulus and uvula contribute to velocity storage, otolith integration, and gravity-referenced internal models of self-motion (Walker et al., 2010; Laurens, 2022; Cullen, 2023). Consequently, cortical tuning changes observed in VIP, MSTd, or PIVC may reflect not only local plasticity, but also the readout, transformation, or contextual modulation of recalibrated signals generated through cerebellar-brainstem circuits.

Taken together, these findings indicate that visual-vestibular recalibration is a distributed and hierarchical process involving interactions among sensory, multisensory, decision-related, and cerebellar networks. Rather than reflecting a uniform shift in sensory encoding, recalibration appears to emerge from coordinated adjustments across multiple levels of the nervous system, providing a neural substrate for the adaptive updating of multisensory perception over time.

### 4.3 Computational frameworks: updating internal models

**FIGURE 6:**
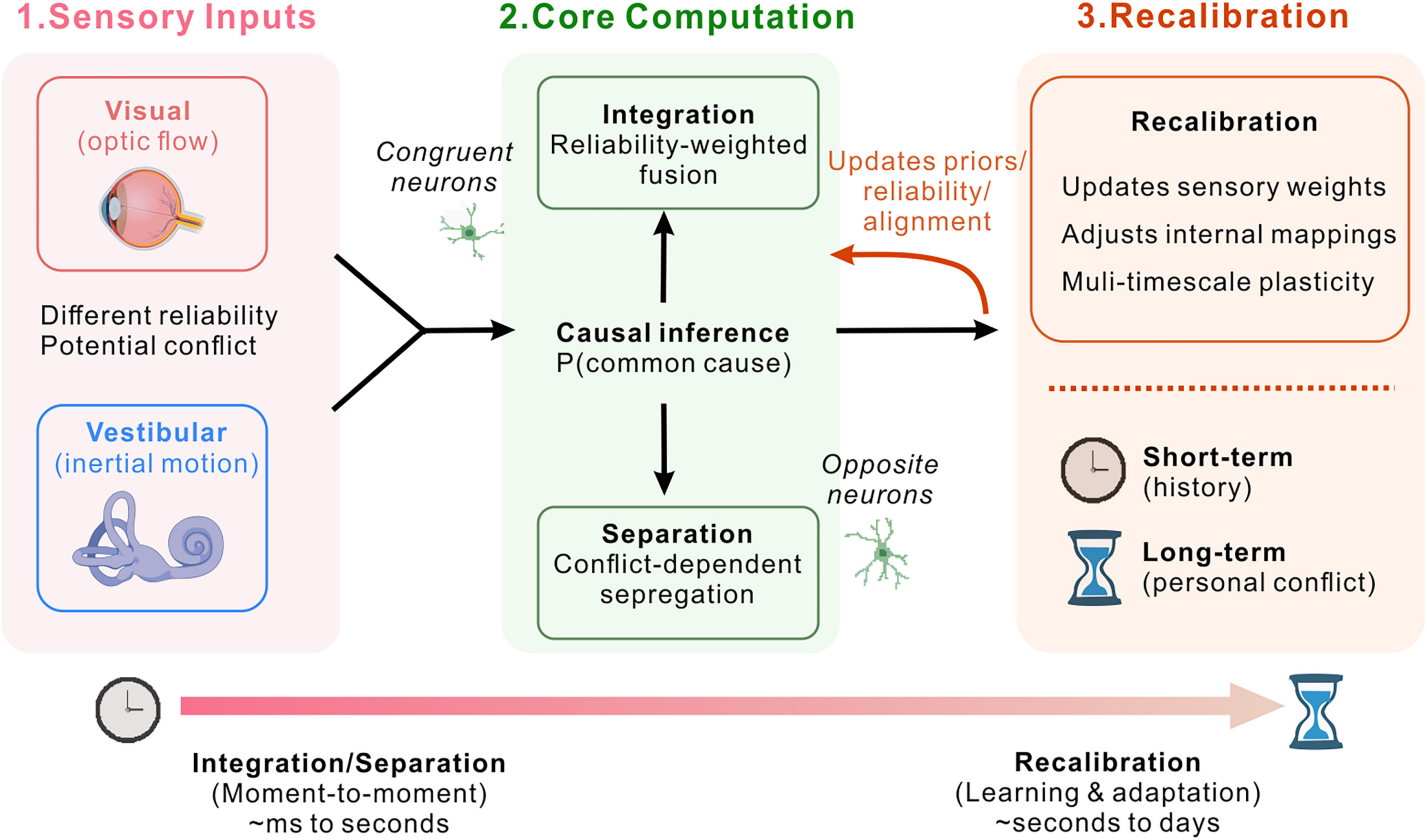
Computational framework linking multisensory inference and recalibration across timescales. Visual and vestibular self-motion signals provide sensory inputs that may differ in reliability and may sometimes conflict. These inputs feed into a core computational stage in which the brain performs causal inference, estimating the probability that the two cues arise from a common cause. A high common-cause estimate favors reliability-weighted integration, whereas a low common-cause estimate favors separation. Recalibration operates on slower timescales to update the internal parameters that shape future inference, including cue weights, cross-modal alignment, and prior expectations. Short-term recalibration reflects recent sensory history, whereas long-term recalibration reflects more persistent sensory conflict and accumulated experience. Together, these processes illustrate how moment-to-moment integration/segregation and longer-term adaptive learning are coupled within a unified computational framework.

From a computational perspective, recalibration can be understood as the process by which the brain updates internal models in response to persistent prediction errors. Within a Bayesian framework, discrepancies between expected and observed sensory inputs lead to updates in estimates of cue reliability, cross-modal alignment, or prior beliefs about causal structure (Kording et al., 2007; Shams and Beierholm, 2010; Rohe and Noppeney, 2015b; Zaidel et al., 2021). As illustrated in Figure 6, this framework links moment-to-moment multisensory inference with slower adaptive changes that accumulate across experience.

Several forms of updating have been proposed. Recalibration may involve shift in sensory mappings, adjusting how signals from different modalities are transformed into a common reference frame (Zaidel et al., 2011). Another perspective emphasizes updating cue reliability estimates, thereby altering how cues are weighted during subsequent integration. More generally, recalibration can be viewed as updating priors over causal structure, influencing the likelihood that cues will be integrated or segregated in future trials (Figure 6). Although these mechanisms differ in implementation, they share the common goal of reducing future prediction errors and improving perceptual accuracy.

Importantly, recalibration naturally extends causal inference models across timescales. While causal inference governs integration and segregation on individual trials, recalibration modifies the underlying parameters-such as priors, mappings, and reliabilities-that shape those inferences over time. In this sense, recalibration provides the mechanism by which short-term inference is consolidated into long-term adaptive change.

More explicitly, this perception-learning loop can be described as a posterior-weighted prediction-error update. On each trial, the discrepancies between visual and vestibular estimates generate sensory prediction error. These errors update internal parameters, such as cue alignment, cue reliability, or common-cause priors, in proportion to both error magnitude and the inferred probability that the cues arise from a common source. Consequently, the same physical conflict may produce different amounts of recalibration depending on whether the brain interprets the cues as belonging together or as arising from separate causes. This prediction provides a direct computational link between causal inference and recalibration.

This Integration-Separation-Recalibration (ISR) framework can also be reformulated within predictive-coding and active-inference accounts. In these models, perception and learning emerge from hierarchical generative models that minimize prediction errors through precision weighting and updating of internal beliefs. Integration reflects the inference of a common cause, segregation reflects competing causal explanations, and recalibration reflects long-term updating of priors, cue reliabilities, or cross-modal mappings when prediction errors persist. Thus, predictive-coding and active-inference frameworks provide an alternative computational language for describing the same interaction between inference and adaptive plasticity.

Visual and vestibular self-motion signals provide sensory inputs that may differ in reliability and may sometimes conflict. These inputs feed into a core computational stage in which the brain performs causal inference, estimating the probability that the two cues arise from a common cause. A high common-cause estimate favors reliability-weighted integration, whereas a low common-cause estimate favors separation. Recalibration operates on slower timescales to update the internal parameters that shape future inference, including cue weights, cross-modal alignment, and prior expectations. Short-term recalibration reflects recent sensory history, whereas long-term recalibration reflects more persistent sensory conflict and accumulated experience. Together, these processes illustrate how moment-to-moment integration/segregation and longer-term adaptive learning are coupled within a unified computational framework.

Computational models also offer interpretations of apparent multi-timescale nature of recalibration. Rather than treating fast and slow effects as separate behavioral observations, these models frame them as constraints on how internal models are updated. One possibility is that recalibration depends on multiple adaptive states with different learning rates, allowing recent sensory history and accumulated conflict to influence perception over distinct temporal windows. Alternatively, apparent timescales may emerge from a single state-space or Kalman-like process in which learning rate is adjusted according to sensory uncertainty, prediction error, and environmental stability (Nassar et al., 2010; Wei and Kording, 2010). In both cases, causal inference can regulate how strongly sensory conflicts drive future recalibration.

Overall, computational accounts suggest that recalibration serves as the mechanism by which transient sensory conflicts are transformed into lasting updates of internal models. By updating sensory mappings, cue weights, and common-cause expectations, recalibration enables the visual-vestibular system to maintain accurate and adaptive self-motion perception under changing environmental conditions.

### 4.4 Translational implications and future directions

Understanding cross-modal recalibration has translational relevance, because many balance disorders involve abnormal resolution of visual-vestibular conflict. Such disorders may arise from impairments in one or more stages of multisensory processing, including abnormal cue weighting, inappropriate integration of conflicting signals, or maladaptive recalibration. For example, symptoms of persistent postural-perceptual dizziness (PPPD) are often aggravated by upright posture, self-motion, and complex visual environments, which may reflect excessive visual dependence, altered visual-vestibular weighting, or impaired recalibration after an initial vestibular event (Dieterich and Staab, 2017; Staab et al., 2017). Similar sensory conflicts occur in virtual reality and cybersickness, where visual self-motion cues are not matched by vestibular signals (Weech et al., 2019). Aging and unilateral vestibular loss provide additional examples in which reduced vestibular reliability leads to increased reliance on visual or proprioceptive information (Alberts et al., 2018, 2019). These processes can be assessed using psychophysical and bedside measures, such as rod-and-frame or rod-and-disk measures of visual dependence and dynamic visual acuity tests of gaze stabilization during head motion (Dannenbaum et al., 2009; Cousins et al., 2014). Such measures may help connect laboratory models of integration, separation, and recalibration with vestibular rehabilitation and clinical assessment.

Future research should combine large-scale neural recordings, causal manipulations, and computational modeling to establish the mechanisms linking integration, separation, and recalibration. A major challenge is to move beyond correlational observations and directly test the causal predictions of the ISR framework. Specifically, ISR predicts that the same physical visual-vestibular discrepancy can produce different perceptual and learning outcomes depending on whether the brain infers a common source or separate causes for the two cues. Candidate regions including MSTd, VIP, and the PIVC/VPS complex could be examined using reversible inactivation, microstimulation, optogenetic perturbation, or activity-guided neural interventions. During visual-vestibular conflict discrimination tasks, perturbing these regions could determine whether they are required for causal-inference-like behavior and for regulating the transition between cue integration and cue segregation. Critical behavioral measures would include changes in perceptual bias, cue weighting, discrimination thresholds, and the disparity boundary at which integration gives way to segregation.

Similar approaches could be applied to recalibration paradigms. Perturbations delivered during prolonged exposure to visual-vestibular conflict could reveal whether specific regions are necessary for converting sensory discrepancies into subsequent adaptive changes. If inactivation or stimulation reduces, abolishes, or reverses post-exposure shifts in visual-only or vestibular-only psychometric functions, this would provide direct evidence for a causal role in recalibration rather than a merely correlational association.

Importantly, the ISR framework makes experimentally testable predictions and is therefore potentially falsifiable. The framework would be challenged if recalibration were fully determined by physical cue discrepancy and cue reliability, independent of common-cause belief or integration-segregation state. Likewise, it would be challenged if cues that are behaviorally segregated produced the same recalibration as cues that are behaviorally integrated. Testing these predictions will help determine whether integration, separation, and recalibration are mechanistically coupled processes or simply co-occurring consequences of multisensory processing.

More broadly, future studies should examine recalibration across sensory modalities, species, and naturalistic environments. Linking multisensory adaptation to decision-making, navigation, and motor control will further clarify how perceptual systems maintain stability while remaining flexible in changing environments. Understanding how distributed cortical, subcortical, and cerebellar circuits cooperate to support these processes remains a central challenge for both basic neuroscience and translational research.

## 5 Conclusion

Visual-vestibular perception operates under uncertainty and frequent sensory conflict, requiring the brain to flexibly determine how cues should be used. Rather than combining signals in a fixed manner, the brain dynamically balances integration, separation, and recalibration.

We propose an Integration-Separation-Recalibration framework in which these processes form a closed computational loop. Integration and separation determine how sensory discrepancies are interpreted on each trial, whereas recalibration uses these discrepancies to update cue weights, cross-modal mappings, and causal priors that shape future inference. Through this loop, moment-to-moment inference and longer-term plasticity are tightly linked.

At the neural level, this flexibility emerges from a distributed cortical network in which distinct neuronal populations and regions contribute differently to cue combination, conflict processing, and adaptive updating. At the computational level, perception reflects the interaction between probabilistic inference and ongoing updates of internal models.

Overall, multisensory perception is not defined by how signals are combined, but by how the brain continuously decides when to integrate, when to segregate, and how to adapt. This framework provides a unifying perspective on perceptual stability and offers a foundation for understanding multisensory computation across contexts and timescales.
